# Melasma in people with darker skin types: a scoping review protocol on prevalence, treatment options for melasma and impact on quality of life

**DOI:** 10.1186/s13643-023-02300-7

**Published:** 2023-08-10

**Authors:** Nomakhosi Mpofana, Buyisile Chibi, Nceba Gqaleni, Ahmed Hussein, Avenal Jane Finlayson, Kabelo Kgarosi, Ncoza Cordelia Dlova

**Affiliations:** 1 Dermatology Department, Nelson R Mandela School of Medicine, University of KwaZulu-Natal, Durban, 4000 South Africa; 2 Department of Somatology, Durban University of Technology, Durban, 4000 South Africa; 3 Center of Rural Health, School of Nursing and Public Health, University of KwaZulu-Natal, Durban, 4000 South Africa; 4 Discipline of Traditional Medicine, University of KwaZulu-Natal, Durban, 4000 South Africa; 5 Faculty of Health Sciences, Durban University of Technology, Durban, 4000 South Africa; 6 Department of Chemistry, Cape Peninsula University of Technology, Bellville, 7535 South Africa; 7 Durban University of Technology, Durban, 4000 South Africa; 8 University of Pretoria, Pretoria, 0002 South Africa

**Keywords:** Melasma, Chloasma, Hyperpigmentation, Hypermelanosis, Treatment, Therapy

## Abstract

**Background:**

Melasma is one of the most encountered dermatoses in dermatology and skin care clinics. It is a challenging chronic, recurrent condition associated with hyperpigmentation. Its aetiology is poorly understood. Melasma affects all races and gender but is more prevalent in women with darker skin types. Being a facial lesion, melasma has a severe impact on quality of life due to its disfigurement. While many modalities of treatment for melasma exists, unfortunately, effectiveness and safety remain a huge concern. Treatment modalities are variable and often unsatisfactory. The objective of this scoping review is to systemically map available evidence from literature regarding melasma on people with darker skin types, garner insight as to how melasma affects the quality of life and begin to investigate and gain understanding on effectiveness of different treatments used for melasma.

**Methods:**

A scoping review guided by Arksey and O’Malley’s framework, the enhancements and recommendations of Levac, Colquhoun and O’Brien, Daudt and associates and the 2015 Johanna Briggs Institute’s guidelines will be conducted. Systematic electronic searches of databases and search engines will include Scopus, PubMed, CINAHL Complete, Cochrane, Science Direct, and Web of Science which will be conducted to attain published peer-reviewed articles of all study designs excluding reviews and grey literature. All literature that meets the inclusion criteria, research question and sub-question will be included in this review. All the retrieved literature will be exported to an Endnote X20 library. Quality appraisal of the included articles will be conducted using the mixed methods appraisal tool (MMAT) 2018 version.

**Discussion:**

We anticipate mapping relevant literature on the melasma, investigating the effectiveness of treatment options of melasma as well as evaluating its association with quality of life in people with darker skin types. This study is likely to reveal research gaps, which could guide future implementation research on melasma treatment interventions.

**Systematic review registration:**

This protocol has been registered a priori with OSF and is accessible on this link: https://osf.io/ru3jc/.

**Supplementary Information:**

The online version contains supplementary material available at 10.1186/s13643-023-02300-7.

## Introduction

Melasma remains a huge medical burden. Melasma is one of the most common hyperpigmentation skin disorders seen more frequently in pigmented skin individuals (Black, mixed ancestry, Indians, Hispanics and African Americans) [1–3]. In a study conducted in South Africa in the public sector by Dlova et al., pigmentation disorders are in the top 5 skin conditions seen, with melasma accounting for most of the cases [4]. While this skin disorder is common among women, men also get affected [5, 6]. There is no known cure for melasma, and currently, available treatment is meant to either halt, stabilize or improve the appearance and progression of the condition [7–9]. The current standard treatment for melasma includes the use of chemical peels, lasers and lights, oral, topical approaches, with hydroquinone serving as the gold standard as well as triple combination creams (includes corticosteroid, tretinoin and hydroquinone) [6, 10–13]. Some of these therapeutic modalities have side effects like contact dermatitis and ochronosis, especially if used for long periods at higher concentrations without proper monitoring by dermatologists. Due to its complex pathogenesis, melasma is challenging to treat and can be quite disfiguring. There is a convincing body of knowledge showing evidence of a negative impact on the quality of life of those affected [14–18].

Concerns about side effects and the long-term safety of current melasma interventions have spurred efforts to investigate alternative treatment options [8, 9, 15, 17]. In rural arrears, black African women use, among others, indigenous plants, for example, *Cassipourea flanaganii* in an attempt to treat hyperpigmentation disorders [19–22]. However, *C. flanaganii* is rapidly becoming extinct as it is highly sought after by the communities [19, 20, 22–24]. It is known that prolonged use of skin lightening products has been proven to be a significant problem for public health as such use can lead to therapeutic failure and drug resistance which could result in death [25, 26]. Most of these ingredients are harmful and pose a health risk considering the frequency of application, the duration of practice, the area of the body involved, their use during pregnancy and lactation put their developing foetus or infants at high risk [27, 28]. Likewise, prolonged use of *C. flanaganii* may pose health risks similar to skin lightening products.

The aim of this scoping review is to systemically map available evidence from literature regarding melasma, garner insight as to how melasma affects the quality of life and begin to investigate and gain understanding on effectiveness of different treatments used for melasma on people with darker skin types. Worldwide, the true prevalence of melasma is unknown hence melasma is viewed as a cosmetic problem, and most patients prefer to consult their dermatologists privately, due to the low recorded prevalence of melasma in most public dermatology clinics in SA, so there is no true representation. Most therapeutic interventions for melasma are imported, making the costs exorbitant and not accessible to the poor of the poorest, resulting in abuse of unsolicited over the counter banned skin bleaching creams. Additionally, there has been reported and undesired side effects more especial when dealing with people of darker skin types. The insights gained from this study could help melasma patients by adding into the treatment armamentarium. A possible multidisciplinary approach may be identified which may help provide new insights both in patients and skin care specialist experience. This scoping review on melasma on people with darker skin types may aid in the future planning and delivery of limited health care resources by focusing dermatology and public health attention on melasma. Melasma may thus be prevented or treated early to limit its progression. Most therapeutic interventions for melasma are imported, making the costs exorbitant and not accessible to the poor of the poorest, resulting in abuse of unsolicited over the counter banned skin bleaching creams. Additionally, there has been reported and undesired side effects more especial when dealing with people of darker skin types. The insights gained from this study could help melasma patients by adding into the treatment armamentarium. A possible multidisciplinary approach may be identified which may help provide new insights both in patients and skin care specialist experience.

## Methodology

The purpose of this scoping review is to systemically map available evidence from literature regarding melasma on people with darker skin types, garner insight as to how melasma affects the quality of life and begin to investigate and gain an understanding of the treatments used for melasma. Furthermore, insights gained from this scoping review will enable and generate a deep scientific understanding when dealing with melasma and may help enhance the current melasma treatment armamentarium.

The research question that guides this scoping review is:

*What effect does melasma have on people with darker skin types and what information is available on efficacy of various treatment interventions?*

Sub research questions are:

1. *What information is available on the type of treatment options available, their efficacy and side effects?*
2. *For those people with darker skin types, what is the prevalence of melasma and the effect on quality of life?*

**Fig. 1:**
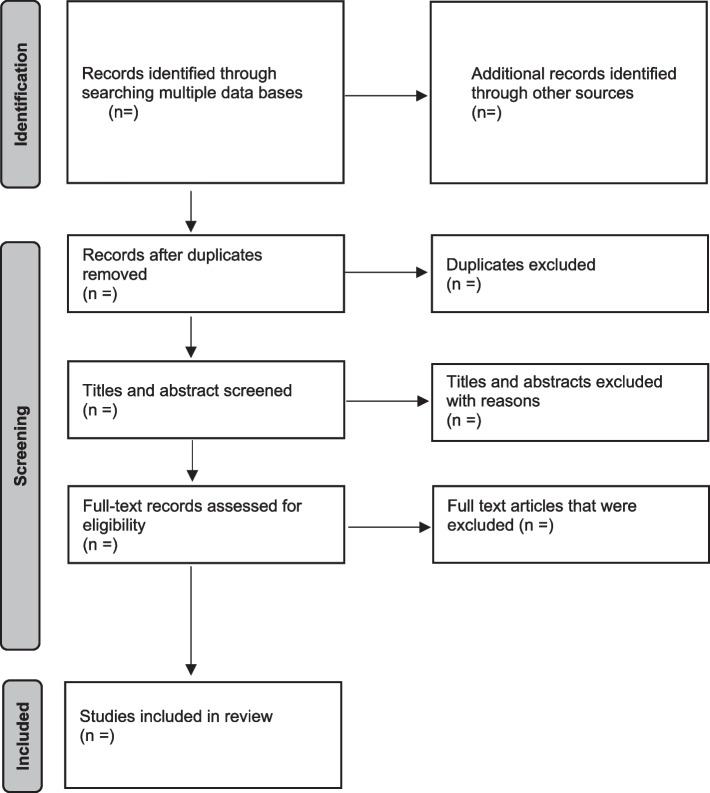
PRISMA-ScR flow diagram demonstrates the literature search and study selection process [32]

To answer the question, a scoping review will be conducted. Guided by Arksey and O’Malley’s framework [29] enhanced by Levac et al. [30] and the 2015 Joanna Briggs Institute’s guidelines [31], evidence in the literature regarding melasma will be mapped. In accordance with this methodological framework, the systematic search will include the six-stage framework as follows: (1) identifying the research question; (2) identifying relevant studies; (3) study selection; (4) charting the data; (5) collating, summarizing, and reporting the articles; and (6) consultation (knowledge translation). To ensure no steps are omitted, the Preferred Reporting Items for Systematic Reviews and Meta-Analysis: Extension for Scoping Review guidelines (PRISMA-ScR) will guide the process [32] (Fig. 1). Furthermore, the results will be summarized and presented using the Preferred Reporting Items for Systematic Review and Meta-Analysis Protocols (PRISMA-P) 2015 checklist to ensure a rigorous process [33].

A systematic search to synthesize literature for published and unpublished records and articles will be undertaken. The Preferred Reporting Items for systematic Reviews and Meta-analyses (PRISMA) guidelines and the Population, Exposure, and Outcome (PEO) framework for determining the eligibility of the research question (Table 1) will be followed. Since the study will utilize a secondary synthesis of data, ethical approval and consent to participate in the study are not necessary as evidence is already available in the public domain.

**Table 1:** PEO framework

Criteria	Determinants
Population	People with darker skin types with melasma
Exposure	Any kind of treatment for melasma
Outcome	(1) What is the extent of the effect of treatment on melasma outcomes. The magnitude of the effects will be measured by extent of changes in the mean scores of all tools used to assess treatment efficacy (2) Extent of reported toxicity or (3) Score on quality of life scale using Melasma Quality of Life measurement scale (MELASQoL)

### Framework stage 1: identifying the research question

The main research question to guide this scoping review study is:

What effect does melasma have on people with darker skin types and what information is available on efficacy of various treatment interventions?

Sub research questions are:

3. What is the prevalence of melasma?
4. How does melasma affect the quality of life of people?
5. What treatment interventions are used to treat melasma?
6. Are there any acceptable, safety systematic profile after a long-term use of these treatments?

A Population, Exposure, Outcome (PEO) framework will be applied to adequately address the research question and eligibility of selected and included literature (Table 1). For population, we will include studies done on people with darker skin types, both male and female, who are classified as Fitzpatrick skin types IV–VI, who suffer from facial melasma. For exposure, we will include studies of treatments used for melasma, and for outcome, we will look at efficacy of treatment, extent of reported toxicity and a possible improvement in quality of life. Types of treatment interventions used for melasma include medical and non-medical interventions. Studies could be published in any language and will not be limited by time frame to ensure that we cover the breadth and comprehensiveness of the available literature. Studies with no evidence of melasma treatment interventions will be excluded as well as studies that investigated extra-facial melasma. The range of evidence will include different methodologies from qualitative to quantitative in nature. Sources such as grey literature and unpublished theses and dissertations will be excluded due to absence of peer review.

### Framework stage 2: identifying relevant studies

Search terms will be applied in a comprehensive electronic literature search which includes the following databases: Systematic electronic search of databases and search engines of Scopus, PubMed, Cochrane, CINAHL Complete, ScienceDirect and Web of Science Core Collection will be conducted to attain published peer-reviewed articles of all study designs excluding reviews. This literature search will not be restricted by language or date of publication. All studies included in the review will be amalgamated and stored using the Endnote × 20 library, and all duplicates will be removed using the “remove duplications” function [34]. We will work closely with the librarians from University of Pretoria and Durban University of Technology during database searching and retrieval of articles. A search strategy using Boolean terms “AND” and “OR” to separate search words and terms as well as MeSH terms and keywords will be used. The search results with keywords, search string, Boolean terms, databases and the number of articles retrieved will be recorded on an electronic database search recording table (Table 2). To obtain additional literature, a hand search of included articles reference lists will be conducted.

**Table 2:** Results of pilot search in PubMed

Date of search	Electronic database	Keywords searched	Number of publications retrieved
12/10/21	PubMed	((“melanosis”[MeSH Terms] OR Melasma [Text Word] OR melanosis [Text Word] OR “skin pigmentation”[MeSH Terms] OR Skin Pigmentation [Text Word] OR dyschromia [Text Word] OR “hyperpigmentation”[MeSH Terms] OR hyperpigmentation [Text Word] OR Chloasma [Text Word] AND (humans[Filter])) AND ((“Quality of Life”[Mesh]) OR “Prevalence”[Mesh] OR “quality of life”[TW] OR prevalence[tw] OR “therapy”[Subheading] OR “therapeutics”[MeSH Terms] OR treatment[Text Word] AND (Fitzpatrick skin IV to VI[TW] OR Fitzpatrick skin IV—VI[TW] OR Fitzpatrick VI[TW] OR Fitzpatrick IV[TW] OR Black[TW] OR Indian[TW] OR “mixed ancestry”[TW] OR “darker skin type*”[TW] OR “African Continental Ancestry Group”[Mesh] OR “mixed race”[tw]	888

We have conducted a pilot search in PubMed in accordance with the inclusion criteria to demonstrate the feasibility of answering our research question using a scoping review method. The results of our pilot search are presented in Table 2. To reduce any selection bias, screening of study titles and abstracts from the databases listed above will be conducted by two investigators independently. All eligible records retrieved from database search will be exported to the endnote library. Using Google forms, the two reviewers (primary and secondary authors) will review in parallel all abstracts applying the inclusion criteria to determine eligibility of the selected and identified records. Google forms will also be piloted to ensure all screeners understand how to utilize the tool. Both reviewers will subsequently conduct full-text screening of all eligible records. Discrepancies between the two reviewers will be resolved through discussion and by inviting a third reviewer. To determine the level of agreement between the two reviewers, the kappa statistics will be used [35]. A kappa statistic of > 0.21 will be considered acceptable agreement while > 0.61 will be considered as an adequate agreement and 0.81–0.99 will be considered as almost perfect agreement.

The search terms (Table 3) will include “melanosis”, “Melasma”, “skin pigmentation”, “dyschromia”, “hyperpigmentation”, “Chloasma”, “Fitzpatrick skin IV to VI”, “Fitzpatrick skin IV – VI”, “Fitzpatrick VI”, “Fitzpatrick IV”, “Black”, “Indian”, “Mixed ancestry”, “darker skin type*”, “African Continental Ancestry Group”, “mixed race”, “quality of life”, “prevalence”, “therapy”, “therapeutics”, “treatment”. Boolean terms, AND or “OR”, will be used to separate the key words, for example: Melasma OR pigmentation OR dyschromia OR hyperpigmentation OR Chloasma AND “quality of life” OR prevalence OR therapy OR therapeutics OR treatment. Each search term will be documented in detail showing the date of search, search engine, key words used and number of publications retrieved (Table 2).

**Table 3:** Search terms

Topic	Population	Exposure	Outcome
Melasma, melanosis, skin pigmentation, dyschromia and chloasma	Fitzpatrick skin IV to VI, Black, Indian, Mixed ancestry, people with darker skin types, African continental Ancestry group, mixed race	Therapy, therapeutics, treatment	Quality of life, prevalence

### Framework stage 3: study selection

Articles and records will be regarded as either eligible for inclusion or exclusion if they meet the following criteria:

#### Inclusion criteria

- Articles that explore facial melasma
- Articles that investigate treatment interventions used for facial melasma
- Articles that explore prevalence of facial melasma
- Articles that investigate effects of facial melasma on quality of life
- Peer-reviewed articles
- No limitation in terms of time frame
- No limitation in terms of language

#### Exclusion criteria

- Articles with no evidence of facial melasma
- Articles focusing on other hypermelanosis disorders other than melasma will be excluded
- Articles that do not test the efficacy of melasma treatment interventions will be excluded
- Articles with no evidence of effects of facial melasma on quality of life
- Review studies
- Grey literature

### Framework stage 4: charting the data

Data charting form will be designed using google forms (Table 4). Data chart form will be independently populated electronically by the screeners with all literature that possesses characteristics and the key information relevant to the review question. The data chart form will be piloted by the two independent reviewers using a random sample of 5 included articles for consistency. The results will be presented as a “map” of the data in a logical table form that aligns to the research question and scope of the review. During this iterative process, the data charting form will be kept current by regularly updating it to ensure accuracy and rigour.

**Table 4:** Data charting form

Data chart heading
Title of study
Author and year of publication
Keywords
Study location
Study sector/setting
Study Aim
Study design
Study population (Fitzpatrick skin types, ethnicity, gender, mean age, severity of melasma)
Types of data sources included
Reported prevalence
Reported treatment interventions
Reported impact on QoL
Reported on clinical features
Reported challenges or limitations
Conclusion

### Framework stage 5: collating, summarizing, and reporting the results

This process comprises of three stages: (1) descriptive, thematic, and quantitative analysis; (2) reporting the results; and (3) identifying literature gaps for future research. The themes from the extracted data will be examined in relation to the aim of the study which is to map evidence from literature on effects of melasma and glean available information on efficacy of various interventions used to treat melasma on people with darker skin types. A narrative report will be produced to summarize the extracted data around the following outcomes: interventions used to treat melasma, prevalence of melasma, effects of melasma on quality of life and efficacy. These results will be described in relation to the research question and the context of the overall study purpose. The review results will be published in a peer-reviewed journal and presented at relevant conferences.

### Quality appraisal

To determine the quality of the selected studies, this review will utilize the Mixed Method Appraisal tool (MMAT) version 2018 to appraise all included evidence. This will help avoid any risk of bias and also ensure that all evidence included in the study is appropriate [36]. The MMAT is a critical appraisal tool designed for the appraisal stage of systematic mixed studies reviews, for example, reviews that include both quantitative and qualitative as well and mixed methods studies [36]. According to Hong et al. [36], the MMAT tool permits appraisal of the methodological quality of five categories of studies as follows: (1) qualitative research, (2) randomized controlled trials, (3) non-randomized studies, (4) quantitative descriptive studies, and lastly (5) mixed methods studies. The two screeners will be responsible for assigning ratings such as 100% for high average articles, 75% for above-average articles, 50% average, and 25% for low-quality articles.

## Discussion

This scoping review aims to identify and describe the effects of melasma on those who suffer from it by systematically mapping the evidence available in literature, garner insight on its prevalence and how melasma affects the quality of life and to investigate and gain an understanding of the efficacy of different interventions used for hypermelanosis on people with darker skin types. Numerous treatment interventions such as hydroquinone (HQ), kojic acid, arbutin, chemical peels, micro-needling, lasers and lights are used to manage melasma; however, the effectiveness of these treatment interventions is limited due to their adverse effects [6, 10–13]. Currently, there is no cure for melasma and treatment is meant to be chronic to try and minimize progression. Being a facial disfiguring lesion, many authors have published data showing evidence of a negative impact on the quality of life of those affected by the condition [7–9]. In rural areas, women use plants including *c. flanaganii* in an attempt to resolve hypermelanosis; however, the plant is becoming extinct due to over harvesting [19, 20, 22–24]; also, long-term systematic effects due to prolonged use of this plant are not yet known.

Melasma is reported to be a huge medical burden. It has been highly reported in patients of Hispanic, African American, Arab, South East Asian, East Asian and African descents [1–3]. Despite numerous attempts to resolve the problem, it is evident that the problem still exists. While many modalities of treatment for melasma exist, unfortunately effectiveness and safety remain a huge concern. Treatment modalities are variable and often unsatisfactory. This portrays a lack of ideas when optimal relief cannot be offered.

The insights gained from this proposed scoping review could help enhance the scientific understanding on how to address melasma. Insights gained from the study could assist in commercialisation and in addition create beneficiation for rural communities. Possible outcomes for this study may include the identification of a holistic, safe and cost-effective alternative and desirable treatment plan for melasma which can be locally manufactured in South Africa, therefore adding to the current treatment melasma armamentarium and in turn improving the quality of life. We plan to disseminate the study’s findings in peer-reviewed journals and at conferences that focus on dealing with hypermelanosis and present at both international and national seminars. This study could assist in mapping out literature gaps pertaining to treatment of melasma in people with darker skin types. The results will help offer recommendations on how further research can address and close these gaps.

### Limitations

The study results will be limited by skin types as it only focuses on people with darker skin types. Strict time lines will be adhered to.

### Supplementary Information

**Additional file 1.** PRISMA ScR.

## Data Availability

All data generated or analysed during this study will be included in the published scoping review article and will be available upon request.

## Abbreviations

HQ: Hydroquinone
*C. flanaganii*: *Cassipourea flanaganii*
SA: South Africa
WHO: World Health Organization
PRISMA ScR: Preferred Reporting Items for Systematic Reviews and Meta-Analysis: Extension for Scoping Reviews
PEO: Population Exposure Outcome
MeSH: Medical Subject Heading
MMAT: Mixed Method Appraisal Tool
UP: University of Pretoria
